# *Yersinia pseudotuberculosis* enterocolitis mimicking enteropathic γδ T-cell lymphoma with abnormal clonality

**DOI:** 10.1186/1471-2334-14-42

**Published:** 2014-01-27

**Authors:** Osamu Imataki, Makiko Uemura, Kensuke Matsumoto, Naoko Ishibashi

**Affiliations:** 1 Division of Endocrinology and Metabolism, Hematology, Rheumatology and Respiratory Medicine, Department of Internal Medicine, Faculty of Medicine, Kagawa University, Kagawa, Japan; 2 Postgraduate Medical Training Center, Faculty of Medicine, Kagawa University, Kagawa, Japan; 3 Division of Hematology, Department of Internal Medicine, Faculty of Medicine, Kagawa University, 1750-1 Ikenobe, Miki-cho, Kita-gun, Kagawa 761-0793, Japan

**Keywords:** *Yersinia pseudotuberculosis*, *Yersinia* enterocolitis, Enteropathic γδ T-cell lymphoma malignant lymphoma

## Abstract

**Background:**

*Yersinia pseudotuberculosis* generally infects the gastrointestinal tract and causes enteropathy symptoms suggesting infection. *Y. pseudotuberculosis* infections are often complicated with intraceliac lymphoadenopathy mimicking malignant lymphoma. This is a first case of *Yersinia pseudotuberculosis* enteropathy mimicking enteropathic γδ T-cell lymphoma. This case highlighted the γδ T-cell reaction to *Yersinia* enterocolitis sometimes mimicking malignant lymphoma clinically.

**Case presentation:**

A 72-year-old female was referred to our institute due to abdominal pain with skin rush, fever and diarrhea. Computed tomography (CT) scanning revealed mucosal swelling of the cecum with enlargement of regional lymph nodes. Laboratory data showed elevated CRP (7.74 mg/dL), an increased level of soluble interleukin-2 receptor (sIL-2R 3095 IU/mL), and CD3+ γδ T-cell circulation in peripheral blood and bone marrow (10.9% and 3.9%, respectively). Increased proportions of γδ T-cells supported the diagnosis of malignant lymphoma. Colonoscopy demonstrated hemorrhagic mucosal erosion with partial ulceration, and the subsequent pathological findings at the inflammation site suggested malignant lymphoma histopathology in the colon. These objective findings were entirely consistent with enteropathic γδ T-cell lymphoma. Thereafter, however, the microbiological results of the patient’s stool at admission showed *Yersinia pseudotuberculosis*, and she was diagnosed as having *Yersinia* enterocolitis. All abnormal findings including subjective symptoms were in remission or mitigated within 2 weeks after her onset. Even the γδ T-cell circulation disappeared (0.04% in peripheral blood), and we speculate that those cells were a reaction to the *Yersinia* infection.

**Conclusion:**

In this case, a differential diagnosis included infectious enterocolitis from other immunogenic or malignant diseases. Although a measurement of sIL-2R is critical in differentiating malignant lymphoma in patients suffering with lymph adenopathy, that is not confirmative. This patient’s case indicates that T cells expressing the γδ T-cell receptor might be associated with the acute and late phase reactions, in which T cells play a role in the construction of granulomas and the establishment of sequelae.

## Background

*Yersinia pseudotuberculosis* is a Gram-negative bacillus organism that can cause gastrointenstinal tract infections. *Y. pseudotuberculosis* infections are often complicated with intraceliac lymphoadenopathy in humans. Some cases progress to severe local inflammation with lymph node swelling, mimicking malignant lymphoma. In light of these characteristics, it is occasionally difficult to differentiate *Yersinia* enterocolitis from malignant lymphoma.

Most *Yersinia* infections arise from food contamination derived from water or raw pork. Notably, *Y. pseudotuberculosis* is known to cause the development of pseudotuberculotic mucosal lesions in the intestine or colon. The pathological features are characterized by an infiltration of various inflammatory cells, including lymphocytes and histiocytes, into necrotic granulomatous mucosa with reactive lymphoid hyperplasia.

## Case presentation

**Figure 1:**
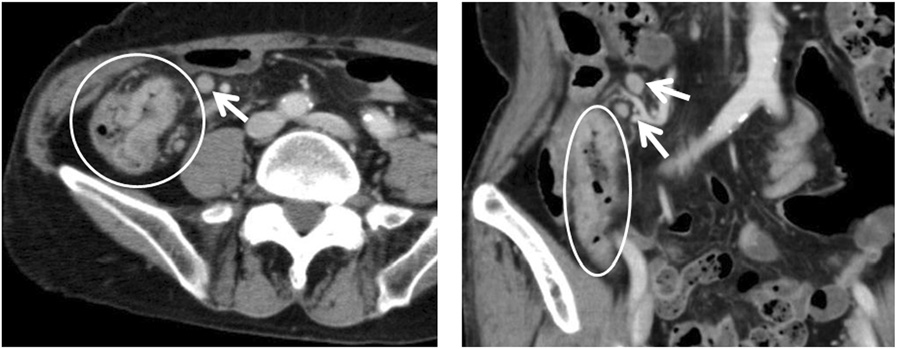
**Her abdominal and pelvic enhanced CT revealed mucosal thickness of cecum (circle) and enlarged lesional lymph node (arrows).** Left panel indicates transverse imaging and right panel indicates coronal imaging.

**Figure 2:**
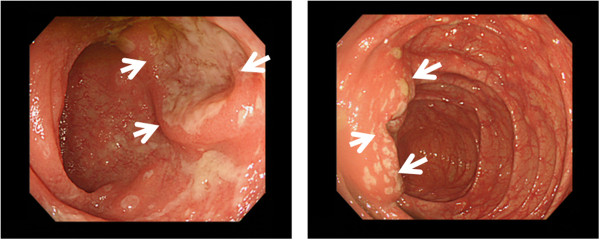
Colonoscopy showed hemorrhagic erosion and ulceration (arrows) at terminal ileum (left) and cecum (right).

**Figure 3:**
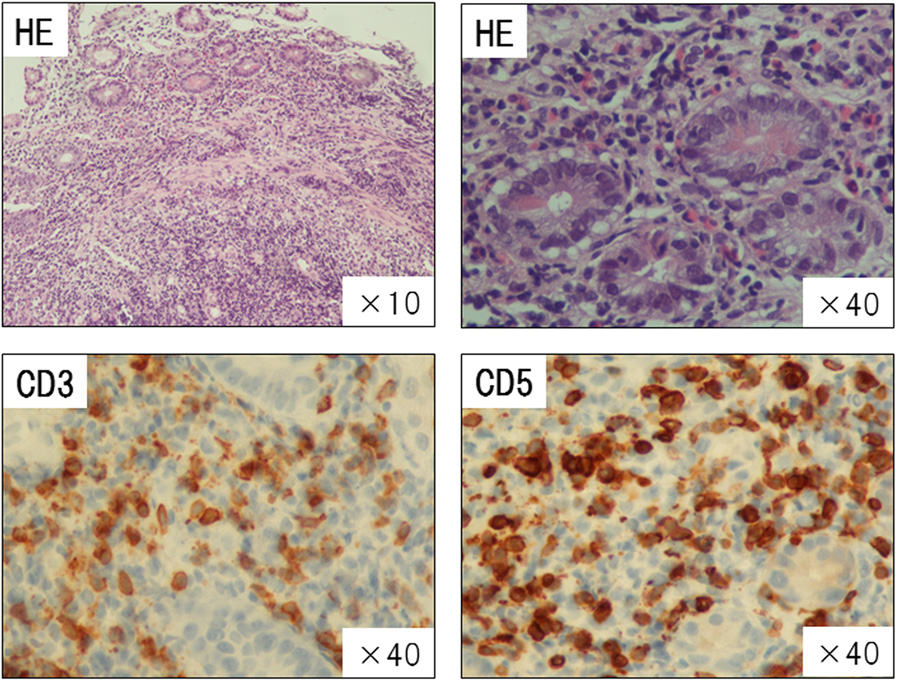
Histopathological findings were compatible of malignant lymphoma of colon mucosa, where small atypical lymphocytes (positive for CD3 and CD5) with cleaved nuclear infiltrate in intra-tubular submucosal area.

A 72-year-old female was referred to our hospital after having experienced fever and abdominal pain for 5 days and a skin rash for 3 days before admission. She also complained of polyarthralgia, predominantly on her distal extremities. At admission, her body temperature was 38.8°C and she had a skin rash composed of exudative maculopapules and erythema multiforme over most of her body. Based on a consultation with a dermatologist who made a tentative diagnosis, we suspended loxoprofen and famotidine, medications she had been taking, because of the suspicion that the eruptions may have been drug-induced. Concomitantly, she received antibiotic therapy (meropenem) under adequate hydration, and underwent repeated blood and stool cultures. Laboratory data showed systemic inflammatory response represented by an increased WBC, 7380/μL, and moderately elevated CRP, 7.74 mg/dL. Her computed tomography (CT) scan demonstrated thickening of mucosa and enlarged mesenteric lymph nodes (Figure 1). A colonoscopy examination showed a round mucosal elevation with hemorrhagic erosion and ulceration at the terminal ileum and cecum (Figure 2), for which the pathological finding was suspicious malignant lymphoma with an infiltration of atypical lymphocytes into necrotic mucosa (Figure 3). Immunohistochemistry determined CD3^+^ T-cell distribution in the submucosal tissue, but did not confirm clonality of lymphocytes. Based on the pathology results, we performed an 18-fluoro-deoxyglucose positron emission tomography (FDG-PET)/CT scan, which drew an accumulation of FDG isotopes into regional mesenteric lymph nodes (SUV max 2.6). In addition, an increased level of soluble interleukin-2 receptor (sIL-2R), 3095 IU/mL, and an expansion of a lymphocyte subset expressing γδ T-cell receptor in peripheral blood and bone marrow (10.9% and 3.9%, respectively) supported the diagnosis of malignant lymphoma. These results led to a tentative diagnosis of enteropathy-associated T-cell lymphoma (EATL, or γδT-cell lymphoma). The patient’s febrile and abdominal symptoms ameliorated rapidly a few days after antibiotics were initiated, and her skin eruptions improved over the following 7 days.

Since we could not obtain a confirmation of the pathological diagnosis, we reevaluated all aberrant clinical findings after the remission of the patient’s symptoms. Within the 2-week treatment course, her laboratory data recovered to normal ranges. The sIL-2R level decreased to 483 IU/mL, and γδ T cells in peripheral blood were almost nonexistent (0.04%). A CT scan and colonoscopy conducted a few weeks after onset revealed slight swelling of the mesenteric lymph nodes and completely normal mucosa. During the second evaluation of the tentative diagnosis, *Yersinia pseudotuberculosis* was identified from her stool culture at admission. In light of all of the findings, we made the final diagnosis of *Yersinia* enterocolitis.

## Conclusion

Most *Yersinia* infection is self-limiting pathogenesis, and in our patient the symptoms improved dramatically immediately after initiation of treatment with a broad antimicrobial, meropenem. Management of *Yersinia* enteroclolits requires antimicrobials and adequate symptom-based supportive care to avoid unfavorable complications such as dehydration. First-line drugs used against the bacterium include aminoglycosides and trimethoprim-sulfamethoxazole (TMP-SMZ). Microbiologically, pathogenesis of *Yersinia* depends on virulence against mucosal tissue that produces a necrotic consequence for the infected host. When encountering a patient with symptoms derived from febrile enterocolitis with or without skin eruption, clinicians should differentiate infectious enterocolitis from other immunogenic or malignant diseases. Causative pathogens of infectious enterocolitis presenting local inflammatory change at the cecum and terminal ileum include *Mycobacterium tuberculosis*, *Salmonella* (*typhi* and *parachyphi*), *Campylobacter*, and *Yersinia*. Invasive suppurative enterocolitis such as *Yersinia* can sometimes mimic other noninfectious pathogenicity, such as autoimmune diseases [1], inflammatory bowel diseases (Crohn’s disease) [2] and malignancies [3]. In this case, a differential diagnosis included infectious enterocolitis from other immunogenic or malignant diseases. A measurement of sIL-2R is critical in differentiating malignant lymphoma in patients suffering with lymph adenopathy.

Over the past 20 years, a wide range of clinical manifestations of *Yersinia* enterocolitis has been revealed, and increased knowledge has been gained about this infection. The mechanism underlying the immunological reaction against *Yersinia*, however, remains obscure. The virulence of *Yersinia* organisms is associated with the enzymatic protein *Yersinia* protein kinase (Ypk), the production of which results in invasive suppurative infection of the lymph nodes [4]. The question of whether specific T lymphocytes are involved in the immune defense against *Yersinia* might be related to the mechanisms underlying this infection’s extraintestinal manifestations and autoimmune-like sequelae, such as erythema nodosum, uveitis, and arthritis.

Autenrieth et al. demonstrated a specific T-cell reaction to *Yersinia* infection in a mouse-infection model [5]. In their phenotypic characterization of reactive T-cell clones on days 14, 20, and 24 after infection of *Yersinia* in C57BL/6 mice, they did not detect T cells positive for γδ T-cell receptors, but all T cells were positive for IL-2R. This result suggests that γδ T cells may no longer have been detectable in a subacute phase reaction at least 14 days after *Yersinia* infection. Autenrieth et al. isolated and identified specific T-cell clones at 12 to 14 days after restimulation by *Yersinia* antigens, and found that these T cells specifically produced a considerable amount of INF-γ but not IL-2. These cells were both CD8 (Lyt2) or CD4 (L3T4)-positive. In fact, CD8 or CD4 T-cell induction by *Yersinia* infection is supported by the same mouse model [6]. In the Bühler study, the authors purified *Yersinia enterocolitica* microparticles, including invasive protein (invasin), and observed both enhanced CD4 and CD8 T-cell responses. They contended that invasion might promote a suitable inflammatory host response, and described the attractive possibility of an invasin vaccination against challenges by other pathogenic entero-organisms.

Although the deficiency of γδ T-cell reaction for *Yersinia* infection was reported in the subacute infection mouse model, Young et al. demonstrated an expression of γδ T-cell receptors 7 to 10 days after stimulation by *Yersinia*-infected autologous B cells [7]. They further elicited the killing ability of generated γδ T-cell lines against *Yersinia*-infected target cells, even though γδ T-cells were generated in concert with autologous synovial fluid mononuclear cells in a major histocompatibility complex (MHC)-independent manner. Young proposed that the participation and contribution of the γδ T-cell response attenuates immunopathological mechanisms of reactive arthritis after *Yersinia* infection. From the Young experiment, we learned that in the early phase, 7–10 days after *Yersinia* infection, γδ T-cells can proliferate and enhance local immunologic reaction in synovia.

In our patient, γδ T cells not only circulated in the peripheral blood but also infiltrated into bone marrow. There may be an effect of γδ T cells on the establishment of tuberculosis-like or lymphoma-like pathology with inflammatory cell responses. Although the immunological role of this T-cell subset phenotypically positive for γδ T-cell receptor is still under investigation, the reaction by these identical T-cell lines to the *Yersinia* organism would explain the multifocal pathology both *in situ* and at extraintestinal sites. This patient’s case indicates that T cells expressing the γδ T-cell receptor might be associated with the acute and late phase reactions, in which T cells play a role in the construction of granulomas and the establishment of sequelae.

## Consent

Written informed consent was obtained from the patient for publication of this case report and any accompanying images. A copy of the written consent is available for review by the Editor of this journal.

## Competing interests

The authors declare that they have no competing interests.

## Authors’ contributions

OI wrote the manuscript and made substantial contributions to the concept and design; MU was involved in drafting and supervising the manuscript; KM suggested important intellectual content and took part in the critical discussion; NI managed the study and reviewed the manuscript; all authors read and approved the final version of the manuscript.

## Pre-publication history

The pre-publication history for this paper can be accessed here:

http://www.biomedcentral.com/1471-2334/14/42/prepub
